# FGFR1β is a driver isoform of FGFR1 alternative splicing in breast cancer cells

**DOI:** 10.18632/oncotarget.26530

**Published:** 2019-01-01

**Authors:** Ming Zhao, Ming-Lei Zhuo, Xiaofeng Zheng, Xiaoping Su, Funda Meric-Bernstam

**Affiliations:** ^1^ Department of Investigational Cancer Therapeutics, The University of Texas MD Anderson Cancer Center, Houston, TX, USA; ^2^ Key Laboratory of Carcinogenesis and Translational Research, Department of Thoracic Medical Oncology-I, Peking University Cancer Hospital and Institute, Beijing, China; ^3^ Department of Bioinformatics and Computational Biology, The University of Texas MD Anderson Cancer Center, Houston, TX, USA; ^4^ Department of Breast Surgical Oncology, The University of Texas MD Anderson Cancer Center, Houston, TX, USA; ^5^ Institute of Personalized Cancer Therapy, The University of Texas MD Anderson Cancer Center, Houston, TX, USA

**Keywords:** FGFR1, alternative splicing, breast cancer

## Abstract

Abnormal FGFR1 alternative splicing is correlated with tumorigenicity and poor prognosis in several tumor types. We sought to determine the roles of FGFR1α and FGFR1β variants in breast cancer. TCGA samples and cell lines were analyzed for FGFR1α/FGFR1β expression. MCF-10A cells were used to overexpress these variants. Cell growth and transformation were assessed by SRB, colony formation, 3D-Matrigel, soft agar, cell motility assays. In TCGA, compared to FGFR1 non-amplified samples, FGFR1-amplified samples had significantly higher FGFR1α but not FGFR1β levels. FGFR1β expression levels and FGFR1β/FGFR1α ratio were higher in basal subtype samples than in ER-positive/luminal samples in both TCGA and breast cancer cell lines. Both FGFR1α and FGFR1β induced transformation of MCF-10A cells. However, only FGFR1β-expressing cells, not FGFR1α, enhanced cell growth and cell motility. Cells with higher FGFR1β levels and FGFR1β/FGFR1α ratio were more sensitive to FGFR inhibitor BGJ-398. Interestingly, in ER-negative cells, FGFR inhibitors decreased FGFR1β levels, likely by increasing expression of splicing repressor PTBP1. In ER-positive cells, estrogen treatment increased FGFR1β levels by decreasing PTBP1 expression, which was blocked by 4-OHT. Lastly, combination treatment with BGJ-398 and 4-OHT synergistically inhibited cell survival. These findings suggest that FGFR1 alternative FGFR1α/FGFR1β splicing plays an important role in breast cancer.

## INTRODUCTION

Breast cancer has high incidence and mortality rate and remains the second leading cause of cancer death in women world-wide [1, 2]. The fibroblast growth factor receptor (FGFR) signaling pathway that regulates cancer cell growth and survival has been reported to play a crucial role in the development and progression of breast cancer [3, 4]. Deregulation of FGFR signaling by genetic alterations of FGFR1 has been found in breast cancer [5–7]. Amplification of FGFR1 located in chromosome 8p11-12 in 10-15% of breast cancer [8, 9], has been correlated with FGFR1 overexpression and poor overall survival, particularly in estrogen receptor-positive (ER^+^) breast cancer [8–10]. Over the past years, alternative splicing events have been discovered in FGFR1 which were implicated in genesis and development of malignant tumors, including breast cancer [6, 11, 12].

Extracellular region of FGFR1 comprises three Ig-like domains, IgI, IgII, and IgIII. Different from two proximal IgII and IgIII domains that determine ligand binding and specificity, the distal IgI and the following linker acid box (AB) sequence are known to have an autoinhibitory function [13, 14]. Alternative inclusion/exclusion of α-exon (exon 3) that covers the IgI and AB linker region creates two splicing forms, FGFR1α and FGFR1β respectively [15–17] (Supplementary Figure 1). This FGFR1 splicing event attracted attention during the past a couple of decades for their differential roles in cancer biology. FGFR1β was reported to have a higher binding affinity to FGF ligands than FGFR1α [18, 19]. Several studies have implicated that overexpression of FGFR1β is associated with tumorigenesis and poor survival in multiple tumors [15, 17–19], while FGFR1α, on the other hand, governs cell differentiation in normal tissues [15, 20, 21], suggesting that the two FGFR1 variants have different effects on cancer cells [19, 20].

In breast cancer, an early study reported that an increased FGFR1β and a decreased FGFR1α expression correlated with reduced survival in breast cancer patients [22]. A recent preclinical study has revealed that FGFR1β promoted breast cancer metastasis while FGFR1α had a suppressing function [23]. Together, these studies suggest that the differential FGFR1 alternative splicing events play an important role in breast cancer. However, given the limited experimental evidence so far, more comprehensive studies are necessary to establish pathophysiological role of alternative splicing of FGFR1β and FGFR1α in governing tumor property of breast cancer and to provide novel therapeutic strategies. The current study was designed for this purpose.

## RESULTS

### Differential expression of FGFR1 splicing variants in breast cancer

**Figure 1:**
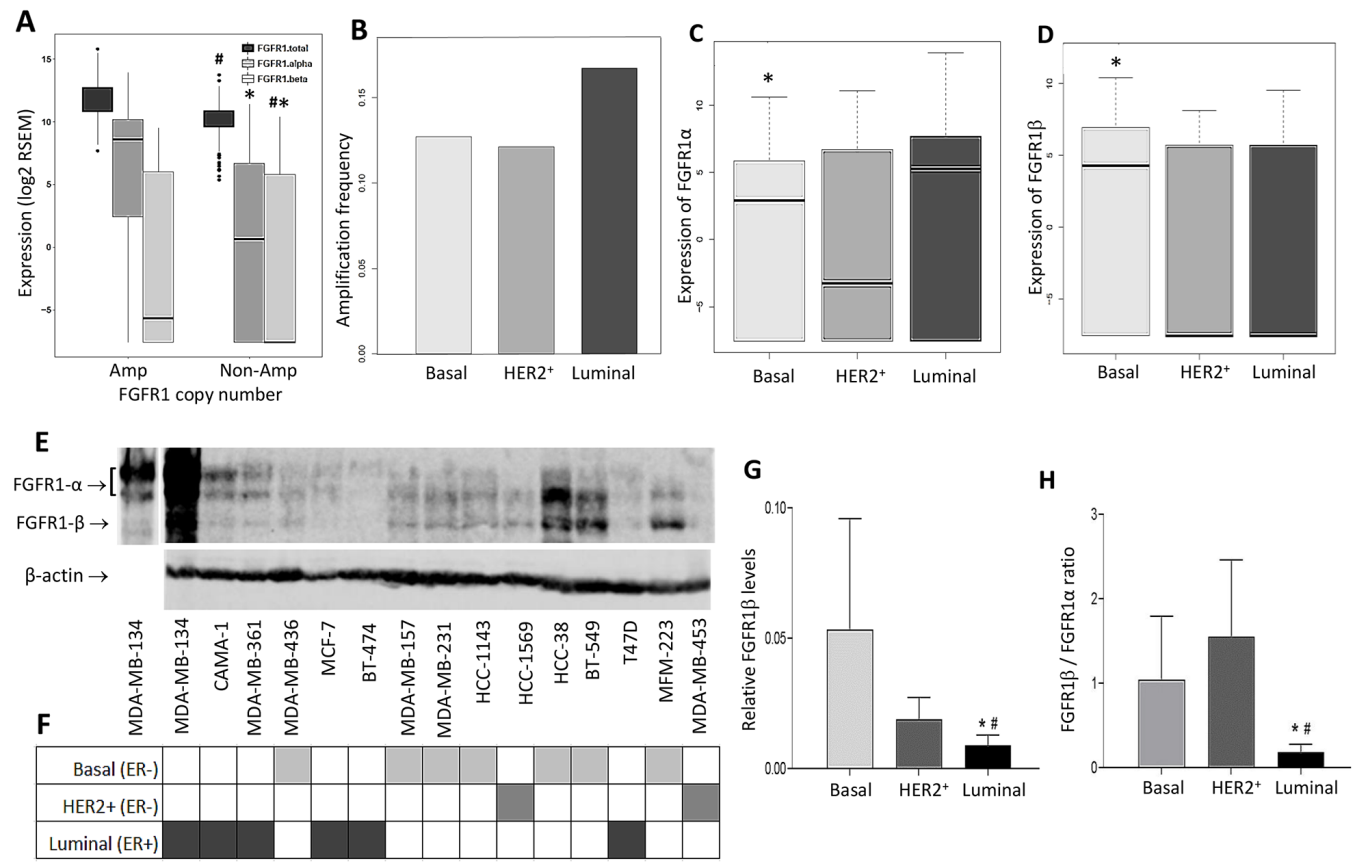
Differential expression of FGFR1α and FGFR1β splicing variants in breast cancer patients and cell lines **(A-D)** Bioinformatic analysis of expression of FGFR1 splicing variants in TCGA breast cancer samples. (A) Expression of FGFR1 variants in FGFR1-amplified and non-amplified samples. ^#^: p=3.69e-19 (amp vs non-amp); ^*^: p=7.35e-15 (amp vs non-amp); ^#*^: p=0.721 (amp vs non-amp). (B) FGFR1 amplification frequency in subtypes. FGFR1 copy numbers were analyzed for amplification frequency in 3 groups of samples – basal, HER2+ and luminal subtypes. (C) Expression of FGFR1α in 3 subtype groups. ^*^: p=0.0305 (basal vs luminal). HER2^+^ vs luminal: p=0.105; basal vs HER2^+^: p=0.669. (D) Expression of FGFR1β in 3 subtype groups. ^*^: p=0.0016 (basal vs luminal). HER2^+^ vs luminal: p=0.812; basal vs HER2^+^: p=0.0725. **(E)** Immunoblotting of FGFR1 in the cell lines. Cell lysates were prepared from 15 breast cancer cell lines and analyzed by SDS-PAGE. FGFR1α and FGFR1β proteins were detected by anti-FGFR1 antibody. The first lane on the left was the same lane of MDA-MB-134VI cells with lighter exposure. **(F)** Subtypes of breast cancer cell lines. **(G)** RelativeFGFR1β levels in cell lines. The relative FGFR1β levels were obtained by normalizing with β-actin. ^*^: p=0.0065 (basal vs luminal); #: p=0.0003 (HER2^+^ vs luminal). **(H)** FGFR1β/FGFR1α ratio in cell lines. FGFR1β and FGFR1α expression levels in WB were quantitated by ImageJ software. The FGFR1-β/FGFR1α ratio was present in each subtype groups. ^*^: p<0.01 (basal vs luminal); #: p<0.001 (HER2^+^ vs luminal).

We analyzed the TCGA database of breast cancer patients and compared expression levels of alternatively spliced FGFR1α and FGFR1β in different breast cancer subtypes. Relative FGFR1α and FGFR1β expression levels were represented by their mRNA expression levels (log2 RSEM) from normalized read counts. First we determined whether both isoforms were overexpressed in patients with FGFR1 amplification. We found that FGFR1α expression levels were significantly higher in FGFR1-amplified samples than that in non-amplified samples (median level 8.6 vs 0.66, p=3.69e-19) (Figure 1A). On the other hand, FGFR1β expression did not exhibit a significant difference between the groups, although its median level was slightly higher in FGFR1-amplified samples compared to non-amplified samples (-5.63 vs -7.56) (Figure 1A). Thus there was a higher FGFR1β/FGFR1α ratio in FGFR1-non-amp samples compared to amplified samples. Next, we examined the expression of FGFR1 isoforms in three breast cancer subtypes. We found that ER^+^ luminal samples had higher FGFR1 amplification frequency than basal and HER2^+^ subtypes (Figure 1B). Interestingly, FGFR1α levels in luminal samples were significantly higher than that in basal subtype samples (median levels 5.29 vs 2.93, p=0.0305) (Figure 1C). In contrast, FGFR1β levels were substantially lower in luminal samples than that in basal samples (median levels -7.56 vs 4.27, p=0.0016) (Figure 1D). No significant differences for expression of either FGFR1α or FGFR1β were detected between luminal and HER2+ and between basal and HER2+ groups (Figure 1C, 1D). The differential expression of these variants leads to a greater FGFR1β/FGFR1α ratio in basal samples than that in luminal samples (p=1.14e-05) (Supplementary Figure 2).

Next, we examined differential expression of FGFR1 splicing variants in breast cancer cell lines. Immunoblot screening of a panel of 15 breast cancer cell lines showed that relative FGFR1β levels were significantly greater in ER^-^ cell lines, particularly in basal subtype, than those in ER^+^ luminal subtype cell lines (Figure 1E–1G, Supplementary Figure 3), which resulted in a consequent higher FGFR1β/FGFR1α ratio in ER^-^ cell lines than in ER^+^ cell lines (Figure 1H). This overall cell line pattern is consistent with the finding in the TCGA patient samples.

### Influence of FGFR1 variants on FGFR signaling and cell proliferation

**Figure 2:**
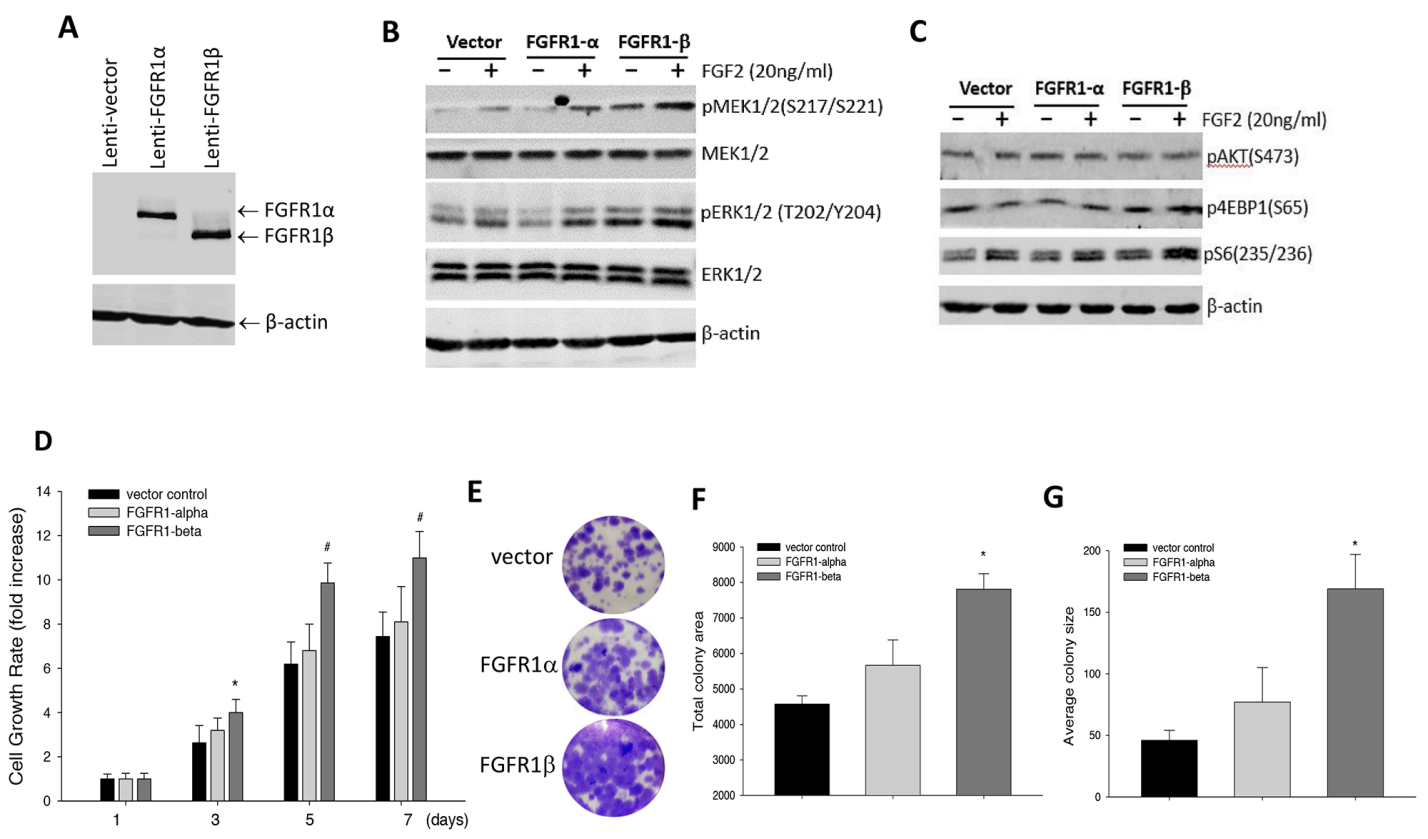
Effects of overexpression of FGFR1α and FGFR1β in MCF-10A cells on FGFR signaling and cell proliferation **(A)** Viral FGFR1 expression. Virus particles were packaged for FGFR1α and FGFR1β, and were infected into MCF-10A cells. Expression of FGFR1 was confirmed by immunoblotting with anti-FGFR1 antibody. **(B, C)** Immunoblotting of FGFR signaling activity. MCF-10Acells were treated with FGF2 at 20ng/ml or vehicle control for 24 hours. The MAPK pathway was detected in the cell lysates with antibodies against phospho-MEK1/2, phospho-ERK1/2 (B), while the PI3K pathway was detected with antibodies against phospho-pAKT, phospho-S6 and phospho-4E-BP1 (C). **(D)** Cell growth rate. MCF-10A cells expressing FGFR1α, FGFR1β, and vector were cultured in 96-well plates in a normal condition for the indicated days. Cell viability was measured by SRB staining. Cell proliferation rate was calculated by normalizing OD490nm values to day 1 OD value. ^#^: p<0.05 vs empty vector; ^*^: p<0.01 vs empty vector. **(E-G)** Cell colony formation. MCF-10A cells seeded in 6-well plates were cultured for 3 weeks followed by crystal violet staining (E). Total colony area (F) and average colony size (G) were quantitated using ImageJ software. Average values were calculated from triplicate wells for each group. ^*^: p<0.01 vs empty vector.

FGFR1 alternative splicing isoforms FGFR1α and FGFR1β have been found to differ in ligand binding. Specifically, FGFR1β has higher binding affinity to FGFs than FGFR1α, and this has been proposed to give rise to more FGF signaling activity in the cells [16, 18, 19]. To evaluate the role of these FGFR1 variants in regulation of FGFR signaling activity, we overexpressed FGFR1α and FGFR1β in MCF-10A cells (Figure 2A). These mammary epithelial cells are known not to express FGFR1 proteins [23]. We analyzed the influence of the FGFR1 isoforms on signaling activities of both MAPK and PI3K pathways. In the MAPK pathway, immunoblotting showed that basal levels of both phospho-MEK1/2 and phospho-ERK1/2 in FGFR1β-bearing cells are substantially elevated above the levels in control cells (Figure 2B). Differently, overexpression of FGFR1α in contrast slightly decreased basal phospho-ERK1/2 levels but mildly increased phospho-MEK1/2 production (Figure 2B). However, there appeared to be no significant difference in response to FGF2 enhancement of signaling activity between all these MCF-10A cells (Figure 2B). In the PI3K pathway, we found that overexpression of FGFR1β increased phospho-S6 in both absence and presence of FGF2, compared to control and FGFR1α-expressing cells (Figure 2C). However, we could not detect changes in phospho-AKT levels between the groups (Figure 2C). FGF2 increased phospho-S6 levels in all three cell lines, without increasing Akt phosphorylation.

Activation of FGFR1 signaling is known to promote cell proliferation. To examine if FGFR1α and FGFR1β play differential roles in cell proliferation, we performed cell SRB growth rate assay. The results showed that overexpression of FGFR1α slightly stimulated cell growth compared to empty vector controls over the time course. But, the MCF-10A cells expressing FGFR1β variant had significantly greater cell growth rate (Figure 2D). To further confirm the effects of FGFR1 variants on cell growth, we assessed colony formation capability of these cells. Similarly, we found that while FGFR1α was able to mildly increase colony formation, FGFR1β-expressing cells exhibited significantly enhanced colony formation capability in comparison with vector controls (Figure 2E). Total colony area and average colony size in FGFR1β-expressing cells were about 2- and 3-fold larger over the control cells (Figure 2F, 2G).

### Influence of FGFR1 variants on cell transformation

**Figure 3:**
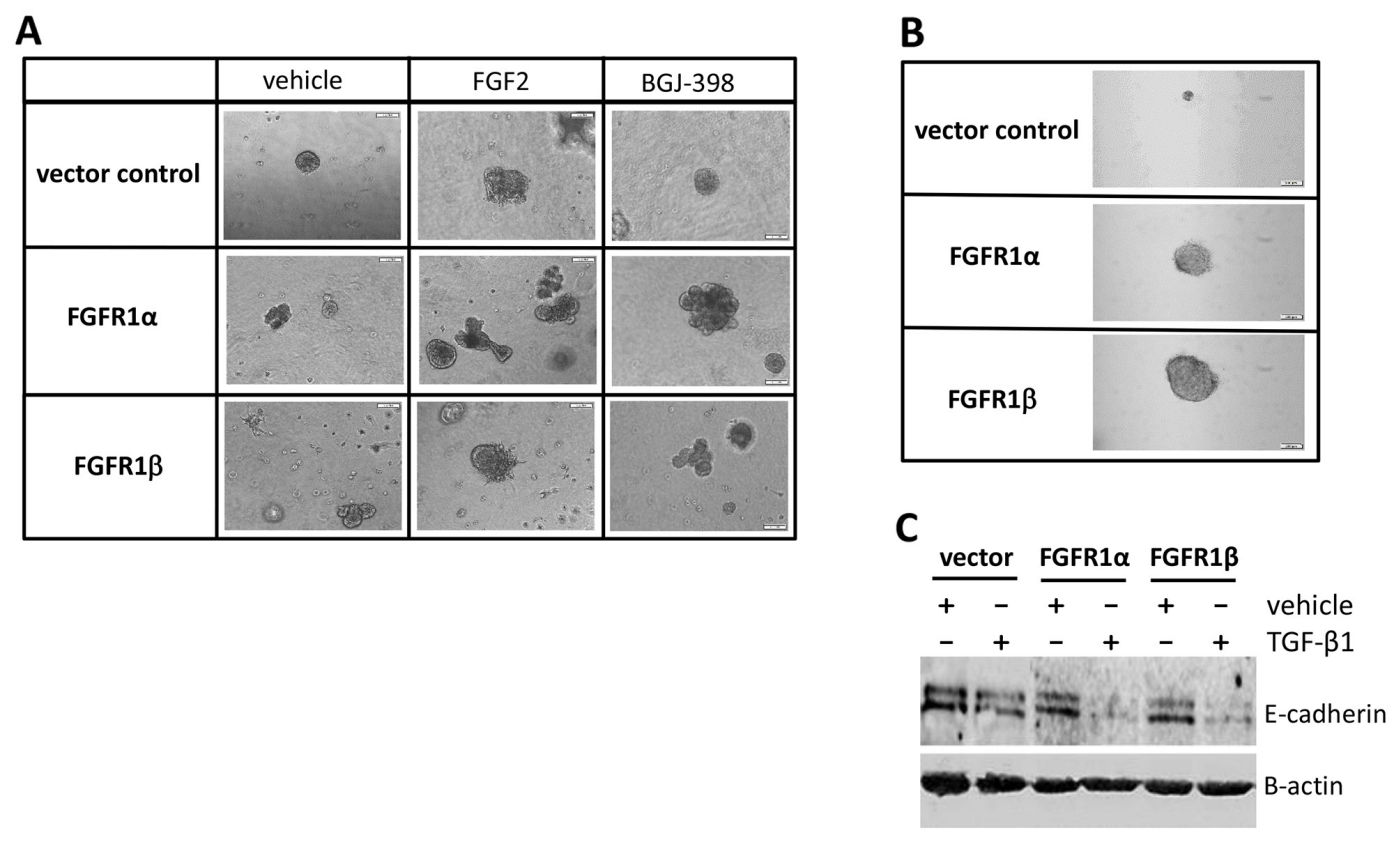
Effects of overexpression of FGFR1α and FGFR1β on cell transformation **(A)** Three-dimensional Matrigel assay. MCF-10A cells expressing FGFR1α, FGFR1β, and vector were seeded into chamber wells coated with Matrigel in MEGM medium containing FGF2 at 20ng/ml and BGJ-398 at 2μM for 3 weeks. Phase-contrast micrographs of spherical mammary structure were captured. **(B)** Soft agar assay. MCF-10A cells were seeded into 3.5% agar gel and cultured for 3 weeks. Anchorage-independent colonies were visualized by Iodonitrotetrazolium chloride staining. **(C)** E-cadherin immunoblotting. MCF-10A cells were treated with TGF-β1 at 5ng/ml or vehicle control for 2 days. Cell lysates were analyzed by western blot using anti-E-cadherin antibody with a normalization by β-actin.

Non-transformed MCF-10A mammary epithelial cells are capable of forming growth-arrested acini-like spheroid architecture on an anchorage independent growth model [24, 25]. To assess the influence of FGFR1 variants on acinar morphology, a three dimensional culture was performed in the presence or absence of FGF2, or BGJ-398 for 3 weeks. Phase-contrast micrograph showed that in the vehicle groups, the control MCF-10A cells processed normal spherical acini-like structure on the 3D-Matrigel. However, expression of FGFR1α and FGFR1β variants disrupted this morphogenetic process, eliciting distinct morphological phenotypes. Both FGFR1α- and FGFR1β-bearing cells formed irregular acinar morphology with abnormal invading protrusions. In some cells, spiculate structures were also observed (Figure 3A). Addition of FGF2 to the culture increased acini size in all three groups, and produced robust and complex multi-acinar structures particularly in FGFR1α-bearing cells, (Figure 3A). FGFR inhibitor treatment appeared not to restore these morphological changes (Figure 3A). In another anchorage independent growth assay, we found that after a three-week culture in soft agar, both MCF-10A-FGFR1α and MCF-10A-FGFR1β cells formed robust colonies, compared to the control MCF-10A cells which formed much smaller colonies in soft agar (Figure 3B).

MCF-10A cells express E-cadherin that plays a principal role in maintaining normal mammary epithelial cell morphology. Disruption of E-cadherin junctions and consequent gain of cell motility contribute to epithelial-to-mesenchymal transition (EMT) [26, 27]. Previous studies demonstrated that TGF-β1 induces EMT of MCF-10A cells by reducing E-cadherin expression [27, 28]. Here, we examined the involvement of FGFR1 splicing variants in this pathological process. Immunoblotting results revealed that overexpression of either FGFR1α or FGFR1β in MCF-10A cells decreased E-cadherin expression and robustly synergized with TGF-β1 treatment to reduce E-cadherin levels (Figure 3C). These results indicate that both FGFR1α and FGFR1β are capable of inducing cell transformation.

### Influence of FGFR1 variants on cell motility

**Figure 4:**
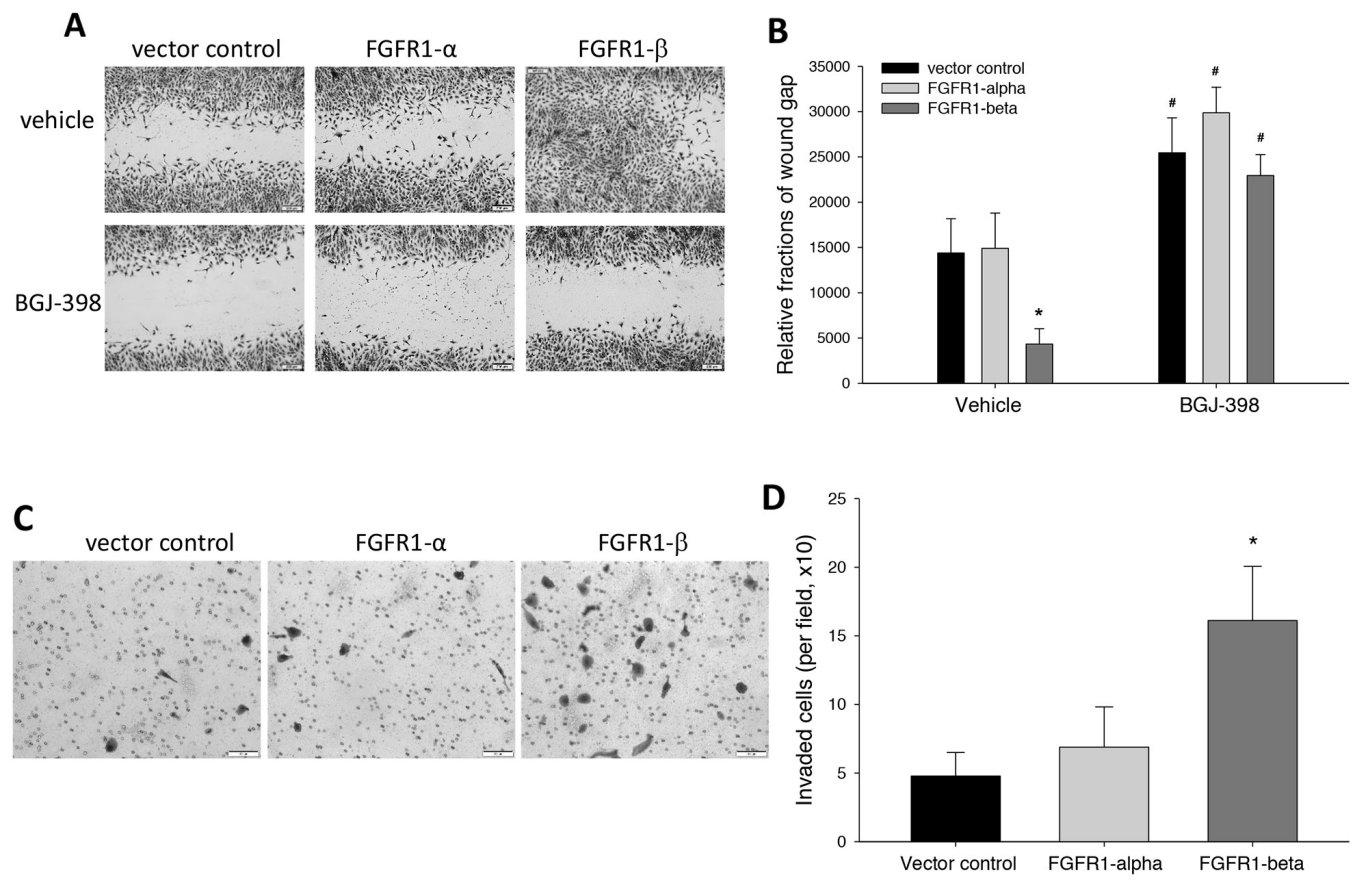
Effects of overexpression of FGFR1α and FGFR1β on cell migration and invasion capability **(A, B)** Wound healing assay. Wound scratches were made on the confluent MCF-10A cells in 12-well plates. Following 2-day culture with BGJ-398 at 2μM or vehicle control for 2 days, followed by crystal violet. Wound gap images were captured (A). Relative wound gap areas were quantitated using ImageJ software (B) ^*^: p<0.01 vs empty vector; ^#^: p<0.01 vs vehicle control. **(C, D)** Transwell matrigel invasion assay. Starved MCF-10A cells were seeded with serum-free medium into transwells coated with basement membrane extract (BME). The transwell inserts were assembled into 24-well plates with 10% FBS medium and cultured for 24 hours. The cells that invaded through the BME were fixed and stained with crystal violet (C). Images of the cells on the membrane were taken. Cell numbers were counted per field (D). ^*^: p<0.001 vs vector control.

Enhanced migration and invasion capabilities are two critical features of transformed tumor cells. Here, we investigated the role of FGFR1 alternative splicing in these pathological events. In wound healing assay, we found that overexpression of FGFR1α did not affect cell migration, compared to control MCF-10A cells, but the FGFR1β-bearing cells migrated vigorously from the edges of wound scratch, leading to almost closure of wound scratch gap, as measured by relative fractions of wound gap (Figure 4A, 4B). Addition of FGFR inhibitor BGJ-398 inhibited cell motility in all three cell groups (Figure 4A, 4B).

We also examined the cell invading ability which is an initial metastatic process of tumor cells to pass enzymatically through the dense surrounding extracellular-matrix of basement membranes and stromal compartments, using a transwell invasion assay. Starved MCF-10A cells were seeded onto the transwells coated with BME and allowed to invade through the BME towards the attraction of serum-containing medium. Staining of the cells that invaded through the BME membrane showed that the cells overexpressing FGFR1β, but not FGFR1α, displayed a higher invading ability than the control cells (Figure 4C). The quantitation of invaded cell number indicated that FGFR1β, but not FGFR1α, significantly enhanced invasion capability of the transformed MCF-10A cells (Figure 4D).

### Influence of FGFR1 variants on downstream functional pathways

We also evaluated the impact of differential levels of FGFR1α and FGFR1β on gene expression of downstream signaling pathways using the same TCGA data. First, we looked at FGFR1α and FGFR1β expressing and non-expressing samples. Out of 18,319 qualified genes, 1512 and 1577 differently expressed genes (DEGs) with FDR (false discovery rate) 0.001 and fold changes larger than 2 were identified to be associated with the differential expression of FGFR1α and FGFR1β respectively (Supplementary Figure 4A,B,G). We also analyzed the samples with differential FGFR1β/FGFR1α ratio. The results of top 10% and bottom 10% FGFR1β/FGFR1α ratio samples showed that among 17,541 qualified genes, 1,777 DEGs were identified to be strongly associated with FGFR1β/FGFR1α ratio (Supplementary Figure 4C,G). In the hierarchically clustered heatmap of DEGs, high FGFR1β/FGFR1α ratio samples and low FGFR1β/FGFR1α ratio samples showed a more distinguishing pattern on separation of DEGs, compared to those for FGFR1α and FGFR1β expressing and non-expressing samples (Supplementary Figure 4A-C). These results indicate that high and low FGFR1α and FGFR1β levels, in particular, high and low FGFR1β/FGFR1α ratios have a largely opposite effects on switching on/off expression of these downstream genes, which may contribute to their divergent tumorigenic function. Furthermore, using Ingenuity Pathway Analysis (IPA) bioinformatics software we performed signaling pathway analysis and identified top 10 canonical pathways for each of DEG analyses (Supplementary Figure 4D-F).

### Influence of FGFR1 variants on cell sensitivity to FGFR inhibitor

**Figure 5:**
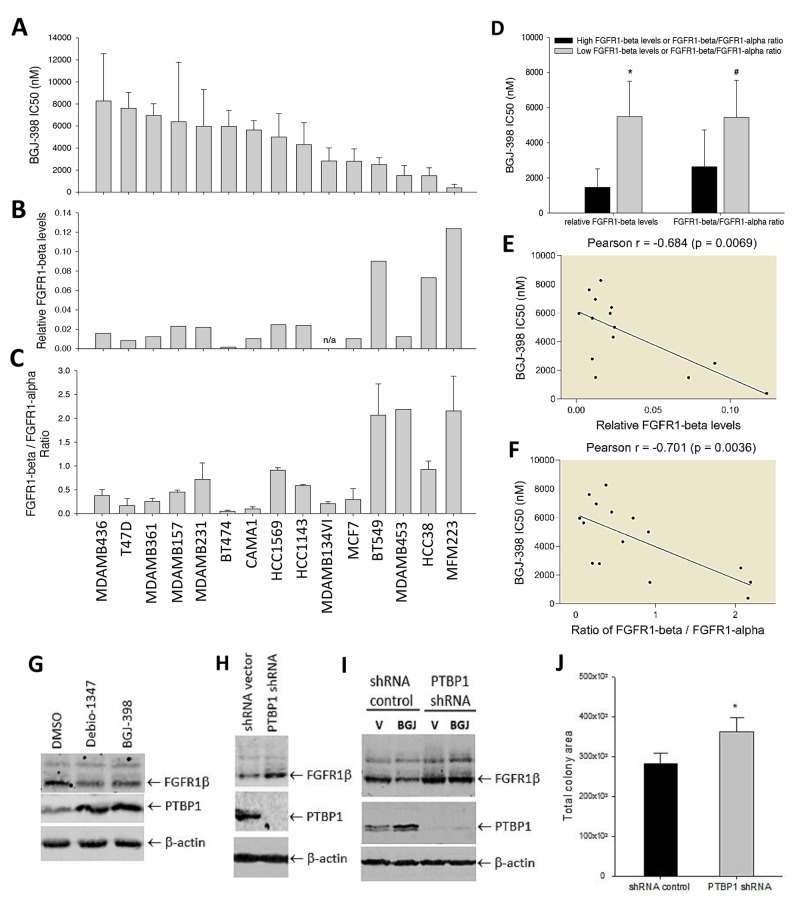
Effects of FGFR1α and FGFR1β on cell sensitivity to FGFR inhibitors on cell survival **(A)** Cell survival screening. Cell lines seeded in 96-well plates were incubated with BGJ-398 at a serial dilutions for 3 days, followed cell viability measurement by SRB. IC50s of cell survival inhibition were calculated using GraphPad Prism7 software. **(B)** FGFR1β levels in cell lines measured by WB in Figure 1C. **(C)** FGFR1β/FGFR1α expression ratio in the cell lines measured by WB in Figure 1C. **(D)** BGJ398 IC50s in cell line groups with high and low FGFR1β levels and FGFR1β/FGFR1α ratio with thresholds 0.05 and 0.9 respectively. ^*^: p=0.015; #: p=0.0033 (low vs high). **(E, F)** Correlations between BGJ-398 IC50 and absolute FGFR1β levels (E) or FGFR1β/FGFR1α ratio (F). The correlation analysis for Pearson r value was performed using GraphPad Prizm7 software. **(G)** MFM-223 cells were treated with Debio-1347 or BGJ-398 at 2uM for 3 days. Expression of FGFR1 and PTBP1 were detected by immunoblotting with anti-FGFR1 and anti-PTBP1 antibodies. **(H)** PTBP1 knockdown. MFM-223 cells were infected with PTBP1 shRNA virus or control shRNA vector. FGFR1 and PTBP1 were detected by immunoblotting. **(I)** PTBP1-deficient cells and control shRNA cells were incubated with BGJ-398 at 2uM or vehicle controls for 3 days, followed by immunoblotting. **(J)** Colony formation of PTBP1-deficient cells. The control and PTBP1-knockdown MFM-223 cells were cultured for 3 weeks for colony formation. Total colony area was quantitated by ImageJ. ^*^: p=0.0136.

We sought to answer if differential expression of FGFR1 isoforms impacts cell response to FGFR inhibitors. Thus, we screened the panel of 15 breast cancer cell lines that differentially expressed FGFR1α and FGFR1β variants as described in Figure 1E-H for their response to BGJ-398 treatment. The differential FGFR1β expression levels and FGFR1β/FGFR1α ratio in these cell lines were displayed in Figure 5B,C. Cell survival assay showed that these cell lines had varying sensitivities to the FGFR inhibitor with IC50s ranging from about 0.3 – 10μM (Figure 5A). Pearson correlation analysis showed that the cell line sensitivity to the FGFR inhibitor was significantly correlated with both FGFR1β levels and FGFR1β/FGFR1α ratio (Figure 5E, 5F). Cells that express high FGFR1β levels and high FGFR1β/FGFR1α ratio were more sensitive to the FGFR inhibitor than the cells with low levels and low ratio (Figure 5D).

We wondered whether FGFR inhibition has an impact on FGFR1 splicing. Surprisingly we found that treatments with FGFR inhibitors BGJ-398 and Debio-1347 were capable of decreasing FGFR1β levels in ER^-^ MFM-223 cells compared to the vehicle treatment (Figure 5G), suggesting that the growth inhibitory effect of the FGFR inhibitors not only results from direct inhibition of FGFR signaling, but may be also a consequence of indirect inhibition of FGFR1β expression. In exploring the potential mechanisms involved in regulation of FGFR1 alternative splicing by FGFR inhibitors, polypyrimidine tract-binding protein 1 (PTBP1) has emerged as a protein of interest, as it is known as a splicing repressor [29–31]. When MFM-223 cells were treated with these FGFR inhibitors for 3 days, they expressed higher levels of PTBP1 than controls (Figure 5G). This action was further confirmed by a knockdown study. We found that in the cells, where the PTBP1 was completely depleted, there was a substantial increase in FGFR1β levels compared to shRNA controls (Figure 5H). Furthermore, we found that PTBP1 loss-of-function clearly removed the effect of BGJ-398 on decreasing FGFR1β expression (Figure 5I). To provide more supporting evidence, we performed chromatin immunoprecipitation (ChIP) assay. The data showed that the splicing regulator PTBP1 bound to one of two specific intronic slicing sequences (ISS) flanking the “α exon” region of the FGFR1 gene in MFM-223 cells (Supplementary Figure 5). PTBP1 deficiency reduced the binding in the cells (Supplementary Figure 5). Functionally, colony formation assay showed that PTBP1 depletion promoted cell growth in these cells (Figure 5J). These results suggest that the FGFR inhibitor downregulates FGFR1β production by upregulating expression of splicing repressor PTBP1 in the cells.

### Estrogen regulation of FGFR1 splicing in breast cancer cells

**Figure 6:**
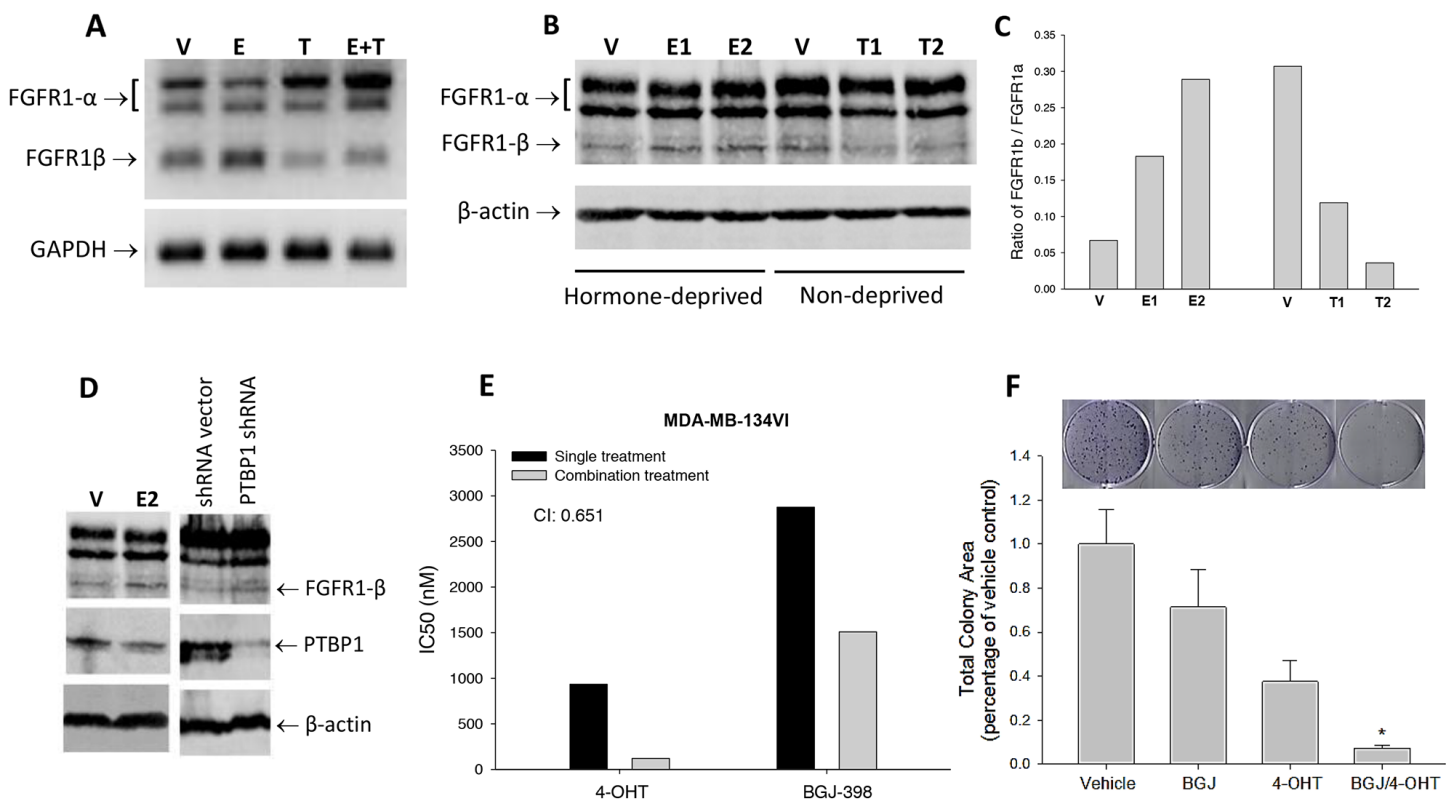
Estrogen regulation of FGFR1 splicing in breast cancer cells **(A)** RT-PCR of FGFR1α and FGFR1β. MDA-MB-134VI cells were cultured for 3 days with hormone-deprived FBS, then treated with 17β-estradiol at 0.1μM (E), 4-hydroxytamoxifen at 1μM (T), or both (E+T), or vehicle (V) for 2 days. RT-PCR was performed to detect mRNAs of FGFR1α, FGFR1β, and GAPDH. **(B)** Immunoblotting. The MDA-MB-134VI cells cultured with hormone-deprived FBS or normal FBS for 3 days were incubated with 17β-estradiol or 4-hydroxytamoxifen treatment respectively for 2 days. E1 and E2: 17β-estradiol at 0.1 and 0.5μM; T1 and T2: 4-hydroxytamoxifen at 1 and 5μM; V: vehicle. **(C)** Ratio of quantitated FGFR1β/FGFR1α expression in Figure 6B. **(D)** WB of FGFR1 and PTBP1. MDA-MB-134VI cells were treated with 17β-estradiol (E2) at 0.1μM or vehicle control for 2 days, or infected with PTBP1 shRNA virus or control shRNA. Expression of FGFR1 and PTBP1 were detected with anti-FGFR1 and anti-PTBP1 antibodies. **(E)** Effects of drug combination on cell survival. MDA-MB-134VI cells seeded in 96-well plates were incubated with single or combination of 4-hydroxytamoxifen and BGJ-398 at doses from 0.05-20000nM and 1250-20000nM respectively for 5 days. Cell survival rate was measured by SRB assay. IC50 and combination index (CI) were calculated using CompuSyn software. (CI<1: synergy; CI>1: antagonism) **(F)** Colony formation assay. MDA-MB-134VI cells were cultured in the presence of BGJ-398 and 4-OHT at 1nM and 0.1nM respectively and their combination for 4 weeks. Colony formation was visualized by crystal violet staining. Total colony area was quantitated using ImageJ.

ER activation is reported to regulate alternative splicing of FGFR2 [32]. Therefore, we were interested in inspecting estrogen regulation of FGFR1 splicing and its consequence in breast cancer cells. First, we examined the expression of FGFR1α and FGFR1β in ER^+^ MDA-MB-134VI cells under the influence of estrogen and its antagonist. RT-PCR results showed that when MDA-MB-134VI cells were exposed to 17-β-estradiol at 0.1μM in a hormone-deprived condition for 2 days, FGFR1α levels were reduced, along with an increase in FGFR1β levels, compared to the vehicle controls (Figure 6A). In contrast, the cells treated with estrogen inhibitor 4-hydroxytamoxifen (4-OHT, the active metabolite of tamoxifen) at 1μM produced less FGFR1α but more FGFR1β variants compared to the controls (Figure 6A). In addition, 4-OHT was also capable of blocking the estrogen-induced changes in alternative FGFR1 splicing (Figure 6A). Consistently, immunoblotting and its quantitation showed that 17-β-estradiol and 4-OHT dose-dependently increased or decreased FGFR1β/FGFR1α ratio respectively in these cells (Figure 6B, 6C). In contrast to FGFR inhibitor, we noticed that 17-β-estradiol treatment clearly reduced PTBP1 levels in MDA-MB-134VI cells, leading to an increase in FGFR1β, compared to vehicle controls (Figure 6D). Similarly, PTBP1 deficiency by shRNA in MDA-MB-134VI cells increased FGFR1β levels (Figure 6D). These results suggest that, in contrast to FGFR inhibitor, activation of ER signaling enhances FGFR1β alternative splicing by inhibiting PTBP1 expression in ER^+^ cells.

Given the fact that both ER and FGFR signaling are involved in regulation of FGFR1β splicing *via* PTBP1, we determined whether there is a synergy between ER and FGFR inhibition on cell survival. First, we found that 17-β-estradiol at 0.1μM increased growth rate of ER^+^ MDA-MB-134VI cells in a time course, while it did not affect ER^-^ MFM-223 cells (Supplementary Figure 6A, 6B). In drug combination study on MDA-MB-134VII cells, we found that co-treatment with ER-antagonist 4-OHT and FGFR inhibitor BGJ-398 substantially reduced IC50s of each drug, compared to the IC50s of single drug treatment, leading to a synergy on cell growth inhibition with a combination index 0.651 (Figure 6E). This synergy was also seen in colony formation assay of MDA-MB-134VI cells where colony formation inhibition was synergistically enhanced by combining BGJ-398 and 4-OHT with a CI 0.78 (Figure 6F). Synergy between 4-OHT and BGJ-398 was also seen in other ER^+^ cells, such as CAMA-1 cells (Supplementary Figure 7A). However, we did not identify synergistic effects between fulvestrant and BGJ-398 (Supplementary Figure 7B, 7C). On the other hand, we also could not detect synergy in ER^-^ breast cancer cells, MFM-223 cells.

## DISCUSSION

Breast cancer has three intrinsic subtypes, basal, HER2^+^, and luminal, based on their gene expression profiles [33]. Results from our bioinformatics analysis of breast cancer patient samples and breast cancer cell line study revealed that FGFR1α and FGFR1β expression have distinct distributions across different groups, including FGFR1-amplified and non-amplified groups, and three subtype groups. In brief, FGFR1-amplified samples have significantly higher FGFR1α expression compared to non-amplified samples, while FGFRβ is not significantly higher. We found that patients with basal tumors express higher FGFR1β levels than luminal breast cancer patients (Figure 1D), which is consistent with the finding from cell lines where FGFR1β levels are higher in basal subtype cell lines than other two subtypes (Figure 1G). However, we could not identify significant differences in FGFR1α and FGFR1β levels between luminal and HER2+ subtypes. This phenomenon may at least in part explain the pathological changes in basal subtype which accounts for up to 90% triple negative breast cancer (TNBC), different from the other two subtypes. Our data suggest that high expression of FGFR1β could be one of crucial risk factors that confer aggressive pathology feature and poor prognosis in basal breast cancer.

Early studies in other tumors have implicated that FGFR1β, but not FGFR1α, plays a pivotal role in tumorigenesis, such as in glioblastoma, astrocytoma, acute myeloid leukemia, and bladder tumor [15, 17–19]. However, in the present study using a mammary epithelial cell model, we found that overexpression of either FGFR1β or FGFR1α in MCF-10A cells is capable of inducing tumorigenic transformation of these normal mammary epithelial cells, as evidenced by formation of irregular spheroid structure in 3D culture and enhanced anchorage independent growth in soft agar. Previous studies found that TGF-β induces epithelial-mesenchymal transition (EMT) of non-malignant epithelial MCF-10A cells by downregulating E-cadherin downregulation [27, 28]. Interestingly, we found that both FGFR1β and FGFR1α synergize with TGF-β-mediated reduction of E-cadherin. This may partially explain why both FGFR1β and FGFR1α similarly induce transformation of mammary epithelial cells. Nevertheless, the basis for the observed differential roles of FGFR1α in tumorigenesis and tumor malignancy between breast cancer and other tumors needs further investigation.

FGFR1 is not only considered important for breast cancer tumorigenesis, but it also has been recently discovered to promote breast cancer metastasis. FGFR1 amplification is more commonly seen in invasive breast carcinoma tissue than in the ductal carcinoma *in situ* (DCIS) [34]. In a knockout mouse model, Wang et al demonstrated that deletion of FGFR1 in mammary tumors greatly reduced tumor metastasis to the lung [35]. Here, we found through *in vitro* invasion and migration assays that FGFR1β, but not FGFR1α, is a dominant FGFR1 isoform that boosts motility of transformed breast cells. This finding is consistent with the phenomenon in Wendth’s *in vivo* mouse model where FGFR1β proved to be required for pulmonary outgrowth of metastatic breast cancer [23].

Abnormal FGFR activity can drive tumorigenesis. Our results demonstrate that FGFR1 alternative splicing variants FGFR1α and FGFR1β function differently in breast cancer. Thus, we wanted to explore the potential mechanisms underlying such distinctive functions. Among a number of identified DEGs associated with FGFR1α and FGFR1β expression levels or FGFR1β/FGFR1α ratio, some are overlapped between these DEG analyses (Supplementary Excel file: DEG FDR0.001). Moreover, IPA assay showed that a number of potential pathways may be involved in these phenotypes, where some pathways are found overlapped (Supplementary PDF file: IPA Pathways). These results may provide speculations to further pursuit the precise molecular mechanisms responsible for the role of FGFR1 splicing in breast cancer.

FGFR signaling is frequently deregulated in many cancers, including breast cancer, playing a role in the pathogenesis and progression of tumors. Therefore, FGFR-targeted therapy could represent a potential therapeutic option for breast cancer patients. Many FGFR inhibitors have been tested in clinical trials on multiple tumor types, including breast cancer [5, 7, 36]. These agents on trials include non-specific FGFR inhibitors Dovitinib, Ponatinib, Lucitanib, Ninedenib, Pazopanib and ARQ and selective inhibitors BGJ398, TAS120, Debio1347, and BAY1163877, AZD4547, JNJ42756493, LY2874455, and PRN1371. However, there appears currently limited single agent efficacy in breast cancer in trials reported to date [37, 38]. There may be several potential reasons for this. FGFR-targeting therapy was proven effective in tumors where FGFR fusions are drivers, such as in cholangiocarcinoma [39–41]. However, FGFR fusions are rare in breast cancer. Previous studies have also suggested other potential drug resistance mechanisms responsible for escape from growth inhibitory effect of FGFR inhibitors, including signaling bypass [42–44]. Therefore, prospective selection of patients with specific FGFR aberrations is one of the major challenges in clinical trials of breast cancer. Among those known deleterious FGFR alterations, the aberrant alternative splicing of FGFR1 reported in this study might be used as a selection biomarker for FGFR-targeting therapeutics. Our *in vitro* data provide supporting evidence for this prospect. For example, breast cancer cells, particularly basal TNBCs, with high FGFR1β levels or a high FGFR1β/FGFR1α ratio are more sensitive to FGFR inhibitors which not only block FGFR signaling activity also reduce FGFR1β expression.

ER has been reported to play an important role in tumorigenic transformation of human breast epithelial cells [45]. While the responsible molecular mechanisms are still under intensive investigation, we found in this study that exposure of ER^+^ cells to estrogen increased FGFR1β expression. Similar to FGFR blockage that decreases FGFR1β levels, ER inactivation by ER antagonist also downregulates FGFR1β expression. Very interestingly, our immunoblotting data from drug treatment and gene depletion experiments demonstrate that regulation of FGFR1β expression by both FGFR and ER signaling share a same mechanism - through the same splicing repressor PTBP1. This novel mechanism provides a rationale for combinatorial therapy with FGFR inhibitors and ER inhibitors, both converging on FGFR1β *via* PTBP1. The synergy of this combination is proven by our *in vitro* testing. In addition, we may also consider combination with agents that modify PTBP1 expression. For example, we found that retinoid acid (RA) was capable of increasing PTBP1 levels leading to reduction of FGFR1β/FGFR1α ratio in breast cancer cells (Supplementary Figure 8). Although PTBP1 is widely known as a repressor in regulation of alternative splicing, some groups reported that this trans-acting protein positively regulates FGFR1β splicing by mediating α exon deletion through specific intronic slicing sequences (ISS1, ISS2) flanking α exon region that encodes the autoinhibition IgI domain [46]. The inconsistency between these reports and our finding however needs to be further clarified. Other than PTBP1, another trans-splicing factor, serine/arginine-rich (SR) splicing factor 55 (SRp55), may also play a role in ER regulation of FGFR1β splicing in ER^+^ breast cancer cells. Jin and Cote identified specific exonic splicing enhancers (ESE1, ESE2) in α exon, and found that interaction between these cis-elements and SRp55 protein controls α exon exclusion [47]. Knocking down SRp55 resulted in more production of α exon-excluded FGFR1 (FGFR1β) than α exon-included one (FGFR1α). Interestingly, it was found that activation of ER downregulated SRp55 levels in ER^+^ MCF-7 cells [48]. SRp55 has been previously implicated in breast cancer, and depletion of SRp55 levels is associated with increased resistance to DNA damage [49]. However, we will pursue examining SRp55 involvement in ER regulation of FGFR1 splicing in the future. In summary, it is expected that in FGFR-targeting therapies of ER^+^ breast cancer patients, simultaneous ER inactivation may produce synergistic therapeutic efficacy by reversing estragon-induced high FGFR1β/FGFR1α ratio in the ER^+^ breast cancer cells.

Although our study has characterized the pathological role of alternative FGFR1 splicing in breast cancer, further study is needed to determin if the FGFR isoforms can assist in idenfifying patients with FGFR-driven breast cancers and help therapeutic decision making.

## MATERIALS AND METHODS

### Analysis of expression of FGFR1 variants in patients

The Cancer Genome Atlas (TCGA) breast cancer level 3 database of total 803 breast cancer samples were used for bioinformatic analysis of expression of FGFR1 and its alternative splicing variants. TCGA BRCA RNA-Seq isoform expression data was downloaded from /rsrch1/bcb/batcheffects/STD_DATA/STANDARDIZED/current/brca/rnaseqv2/illuminahiseq_rnaseqv2_isoform/Level_3. The isoform expression was log2 (RSEM+ point one percentile). The transcript containing exon 3 (Che8:38287200-38287466) that encodes IgI domain is FGFR1α, otherwise is FGFR1β. In amplified FGFR1 samples (copy number ≥ 4) and non-amplified samples (copy number < 2.55), *T*-test was used to test the difference of expression levels of FGFR1, FGFR1α and FGFR1β between amplified and non-amplified samples. Anova and Kruskal Walis rank sum tests were used to test the difference of FGFR1α and FGFR1β expression levels and their ratio respectively between 3 breast cancer subtypes, including basal, HER2-amplified (HER2^+^), and luminal (ER^+^/HER2^-^ and ER^+^/HER2^-^). Out of the 803 samples, top 10% samples with high and low FGFR1β/FGFR1α ratio and top 10% samples with high and low FGFR1β levels were used to identify differentially expressed genes (DEGs). Genes with very low counts (<30 reads) were filtered out, which left 17,541 genes for DEG analysis. Unsupervised hierarchical clustering and principal component analysis (PCA) plot were used for quality assessment. 1,777 DEGs that have FDR 0.01 with fold changes larger than 2 smaller than ½ were identified with generalized linear model (GLM) likelihood ratio test and visualized by heatmap. Association of canonical signaling pathways with DEGs was analyzed using Ingenuity Pathway Analysis (IPA) software (version 470319M).

### Cell lines, drugs and other reagents

A panel of 14 breast cancer cell lines and one non-tumor mammary epithelial cell line were obtained from ATCC, including BT-474, BT-549, CAMA1, HCC-38, HCC-1143, HCC-1569, MCF-7, MMDA-MB-134VII, MMDA-MB-157, MMDA-MB-231, MMDA-MB-361, MMDA-MB-436, MDA- MDA-MB-453, T47D, and MCF-10A. Breast cancer cell line MFM-223 cells were purchased from Sigma. All the breast cancer cell lines were cultured in Dulbecco’s modified Eagle’s medium/F-12 (DMEM) supplemented with 10% fetal bovine serum at 37° and humidified 5% CO_2_. MCF-10A cells were cultured in complete mammary epithelial cell growth medium (MEGM) supplemented with growth factors and insulin (Lonza). To create FGFR1α and FGFR1β variants, MCF-10A cells were infected with virus packaged from 293 cells using viral expression vectors pLenti-C-Myc-FGFR1α (#RC202080L1) and pLenti-C-Myc-FGFR1β (#RC210629L1) plasmids as well as empty vector (#PS100064) (OriGene, Rockville, MD). FGFR inhibitor BGJ-398 was purchased from Selleck Chemicals (Houston TX, USA). 17β-estradiol and 4-hydroxytamoxifen were purchased from Sigma. Growth factors FGF2 and TGF-β1 were purchased from R&D Systems (Minneapolis, MN). BD Matrigel™ Basement Membrane Matrix Growth Factor Reduced was purchased from BD Biosciences (San Jose, CA). Immunoblotting antibodies purchased from Cell Signaling Technology (CST) include anti-FGFR1 (#9740), anti-phospho-ERK_1/2_/T202/Y204 (#4370), anti-ERK_1/2_ (#9102), anti-phospho-MEK_1/2_/S217/221 (#9154), anti-MEK_1/2_ (#9126), anti-E-cadherin (#3195), anti-phospho-AKT/S473 (#4060), anti-phospho-S6/S235/236 (#4858), anti-phospho-4E-BP1/S65 (#9456), and anti-PTBP1 (#57246). Anti-β-actin antibody (#A5441) was purchased from Sigma. Secondary antibodies Goat-anti-Rabbit-Alexa Fluor-680 (#A21076) and Goat-anti-Mouse- Dylight-800 (#610145-121) were purchased from Life Tech and Rockland Immunochemicals respectively.

### Western blot assay

Cells were lysed in 2x Laemmli buffer, followed by protein concentration measurement using Pierce BCA protein assay Kit (ThermoFisher). After SDS-PAGE, proteins were transferred to a 0.2μm nitrocellulose membrane (Bio-Rad Laboratories). Membranes were blocked with 0.1% casein blocking buffer, followed by immunoblotting with the primary antibodies as described in Cell lines, Drugs and Reagents at room temperature overnight. After washing, the membrane was probed with the second antibodies with fluorescence conjugation. The immunoblots were visualized using the Odyssey IR imaging system (Li-Cor Biosciences).

### Cell viability assay

Cells were seeded in 96-well plates at densities of 0.3-1.0 × 10^4^ cells/100μl per well in triplicate for each treatment dose. After adhering overnight, 100μl of drug at serially diluted concentrations were added to the wells and incubated at 37°C for 72 hours. Cells were fixed with 50% trichloroacetic (TCA) followed by staining with 0.4% sulforhodamine B (SRB) solution. OD values were read at 490nm by plate reader Synergy 4 (BioTek). The half maximal inhibitory concentration (IC_50_) was determined using GraphPad Prism v6.05 software.

### Colony formation assay

Cells were seeded in 6-well plates at a density of 500-1000 cells per well in triplicate for each treatment group. Cells were cultured for 3 weeks. Culture medium was changed with fresh drugs twice a week. The cell colonies were fixed in 10% formalin and stained with 0.05% crystal violet in 25% methanol. The stained colonies in the wells were scanned and total colony area and average colony size were quantitated using NIH ImageJ v.1.48 software.

### 3D-Matrigel assay

Cells were seeded into 8-well glass chamber slides coated with growth factor reduced BD Matrigel™ Basement Membrane Matrix at 5000 cells/well in a complete MEGM medium containing 2% Matrigel, in the presence or absence of FGF2 or BGJ-398. Refresh culture medium every 4 days with 2% Matrigel MEBM medium and drugs. Cells were cultured for 2 weeks. Photomicrograph of spherical structures formed on the Matrigel was performed.

### Soft agar assay

6-well plates were coated with 0.5% bottom agar (Difco Agar Noble, BD, #214220). Cells were mixed with 0.35% top agar containing 10% FBS and seeded onto the bottom agar wells at 5000 cells/well. 1ml complete MEGM medium was added on top the agar. Cells were fed twice a week and cultured for 3 weeks. Colonies in the agar were stained with iodonitrotetrazolium chloride (INT) overnight, followed by colony photomicrograph.

### Wound healing assay

When cells cultured in 12-well plates were confluent, a cross scratch was made on the cell layer with 1ml tip. Medium was changed to remove detached floating cells. Cells were cultured with or without BGJ-398 treatment for 2 days to allow cell migration, followed by 10% formalin fixation and crystal violet staining. Photomicrograph of wound gap was performed, followed by quantitation using ImageJ. Relative fraction of wound gap was converted from wound gap area using a formula: wound gap area = (100 / %Area) x Total Area.

### Transwell invasion assay

Transwell inserts (Corning, #354578) were coated with basement membrane extract (BME) Matrigel (Corning, #356234). Starved cells were seeded into the inserts at 5 × 10^4^ cells / insert in 0.3 ml serum-free medium. The inserts were assembled into wells of 24-well plates containing 0.5ml medium with 10% FBS. Cells were cultured for 24 hours. Cells on the up-surface of the inserts were removed by scrubbing with cotton swabs. Cells that invaded through the BME were fixed on the down-surface of the inserts with 10% formalin, followed by staining with 0.4% crystal violet. The stained cells were imaged by inverted microscope at x10 magnification. Cells numbers were counted per image field. 9 fields were quantitated for each group.

### RT-PCR assay

Total RNA was extracted from cell lysates using an RNeasy Mini Kit (Qiagen) and quantitated by Qubit RNA BR Assay (Invitrogen). cDNA was prepared using a High Capacity cDNA Reverse Transcription Kit (Applied Biosystems), followed by PCR using a TaqMan^®^ Universal PCR Master Mix (Applied Biosystems). PCR primers “5’-TTCTGGGCTGTGCTGGTCAC (forward)” and “5’-CTTGTAGACGATGACCGACC (reverse)” were used to amplify FGFR1 variants.

### ChIP assay

MFM-223 cells cultured in 100-mm petri dishes were treated with 1% formaldehyde for 10 min to cross-link chromatin. Chromatin immunoprecipitation (ChIP) assays were performed following the protocol of a ChIP assay kit (Sigma, #17-295). Briefly, the cells were scraped and sonicated on ice to shear chromatin DNA down to 0.2- to 1.0-kb fragments. The sonicated cell supernatant was precleared with a protein A agarose-salmon sperm DNA slurry, and then anti-PTBP1 antibody was added to the supernatant at 4°C overnight with rotation, followed by incubation with fresh protein A agarose beads for 1 h at 4°C for precipitation. The specific protein-DNA complex was reversely cross-linked, and DNA fragments were purified. With these DNAs as templates, PCR were performed to amplify ISS region using primers 5’-caactccggacacaaagaag and 5’-catcacttactggaggctac.

## SUPPLEMENTARY MATERIALS FIGURES







## Abbreviations

FGFR: fibroblast growth factor receptor
TGFβ: transforming growth factor-β
ER: estrogen receptor
PTBP1: polypyrimidine tract-binding protein 1
TNBC: triple negative breast cancer
TCGA: The Cancer Genome Atlas
SRB: sulforhodamine B
BME: basement membrane extract
EMT: epithelial-to-mesenchymal transition
ESE: exonic splicing enhancers
DEGs: differently expressed genes
FDR: false discovery rate
IPA: Ingenuity Pathway Analysis
