# Correction: FGFR1β is a driver isoform of FGFR1 alternative splicing in breast cancer cells

**DOI:** 10.18632/oncotarget.27353

**Published:** 2019-12-10

**Authors:** Ming Zhao, Ming-Lei Zhuo, Xiaofeng Zheng, Xiaoping Su, Funda Meric-Bernstam

**Affiliations:** ^1^ Department of Investigational Cancer Therapeutics, The University of Texas MD Anderson Cancer Center, Houston, TX, USA; ^2^ Key Laboratory of Carcinogenesis and Translational Research, Department of Thoracic Medical Oncology-I, Peking University Cancer Hospital and Institute, Beijing, China; ^3^ Department of Bioinformatics and Computational Biology, The University of Texas MD Anderson Cancer Center, Houston, TX, USA; ^4^ Department of Breast Surgical Oncology, The University of Texas MD Anderson Cancer Center, Houston, TX, USA; ^5^ Institute of Personalized Cancer Therapy, The University of Texas MD Anderson Cancer Center, Houston, TX, USA

**This article has been corrected:** Due to errors during image assembly, the beta-actin control in Figure 6D was accidentally flipped. The corrected Figure 6D is shown below. The authors declare that these corrections do not change the results or conclusions of this paper.

Original article: Oncotarget. 2019; 10:30–44. 30-44. https://doi.org/10.18632/oncotarget.26530

**Figure 6:**
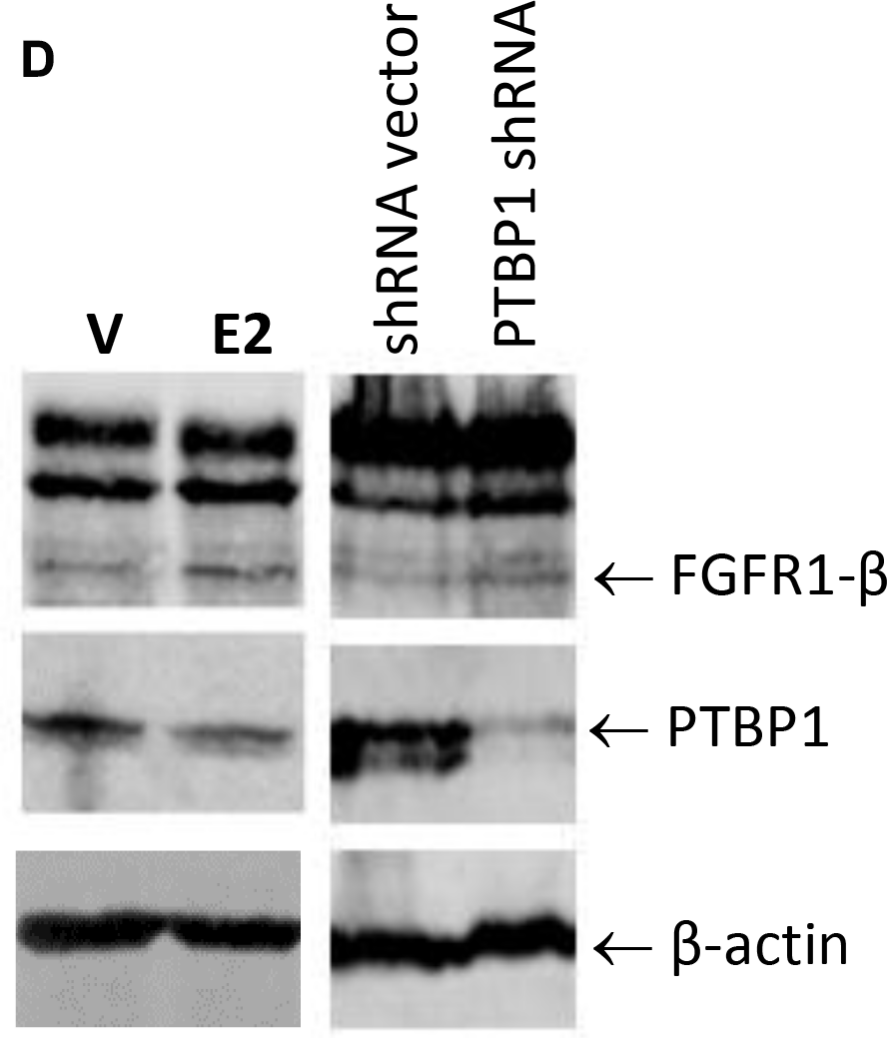
Estrogen regulation of FGFR1 splicing in breast cancer cells.

(**A**) RT-PCR of FGFR1α and FGFR1β. MDA-MB-134VI cells were cultured for 3 days with hormone-deprived FBS, then treated with 17β-estradiol at 0.1μM (E), 4-hydroxytamoxifen at 1μM (T), or both (E+T), or vehicle (V) for 2 days. RT-PCR was performed to detect mRNAs of FGFR1α, FGFR1β, and GAPDH. (**B**) Immunoblotting. The MDA-MB-134VI cells cultured with hormone-deprived FBS or normal FBS for 3 days were incubated with 17β-estradiol or 4-hydroxytamoxifen treatment respectively for 2 days. E1 and E2: 17β-estradiol at 0.1 and 0.5μM; T1 and T2: 4-hydroxytamoxifen at 1 and 5μM; V: vehicle. (**C**) Ratio of quantitated FGFR1β/FGFR1α expression in Figure 6B. (**D**) WB of FGFR1 and PTBP1. MDA-MB-134VI cells were treated with 17β-estradiol (E2) at 0.1μM or vehicle control for 2 days, or infected with PTBP1 shRNA virus or control shRNA. Expression of FGFR1 and PTBP1 were detected with anti-FGFR1 and anti-PTBP1 antibodies. (**E**) Effects of drug combination on cell survival. MDA-MB-134VI cells seeded in 96-well plates were incubated with single or combination of 4-hydroxytamoxifen and BGJ-398 at doses from 0.05-20000nM and 1250-20000nM respectively for 5 days. Cell survival rate was measured by SRB assay. IC50 and combination index (CI) were calculated using CompuSyn software. (CI<1: synergy; CI>1: antagonism) (**F**) Colony formation assay. MDA-MB-134VI cells were cultured in the presence of BGJ-398 and 4-OHT at 1nM and 0.1nM respectively and their combination for 4 weeks. Colony formation was visualized by crystal violet staining. Total colony area was quantitated using ImageJ.

