# Supplementary material for: FGFR1β is a driver isoform of FGFR1 alternative splicing in breast cancer cells
Source: Oncotarget. 2019 Jan 1;10(1):30–44. doi: 10.18632/oncotarget.26530 (PMC6343755; doi:10.18632/oncotarget.26530)
Supplement: Supplementary file 1 [file oncotarget-10-30-s001.pdf]

FGFR1β is a driver isoform of FGFR1 alternative splicing in breast cancer cells

SUPPLEMENTARY MATERIALS

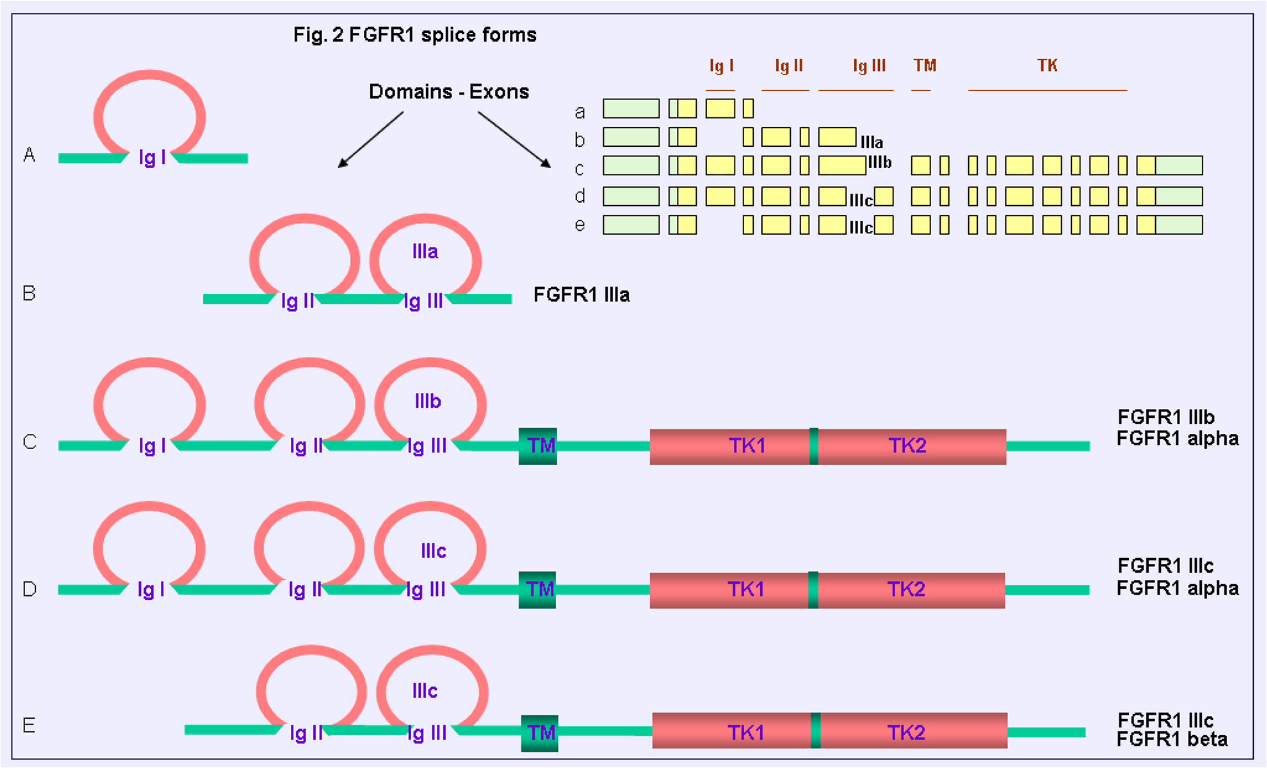

Supplementary Figure 1: Schematic diagram of alternative FGFR1 splicing. IgI, IgII and IgIII: three Ig-like domains. TM: transmembrane domain. TK: tyrosine kinase domain. (http://atlasgeneticsoncology.org/Genes/FGFR1ID113.html).

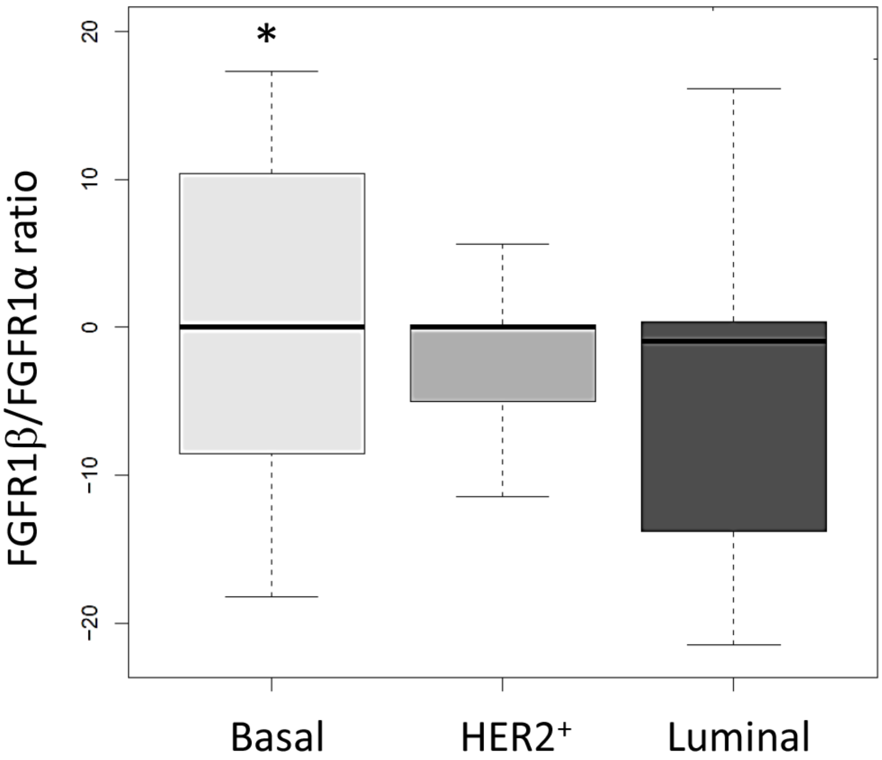

Supplementary Figure 2: Bioinformatic analysis of expression of FGFR1 splicing variants in TCGA breast cancer samples. FGFR1β/FGFR1α ratio in 3 subtype samples. *: p=1.14e-05 (basal vs luminal). $HER2^{+}$ vs luminal: p=0.317; basal vs $HER2^{+}$: p=0.142.

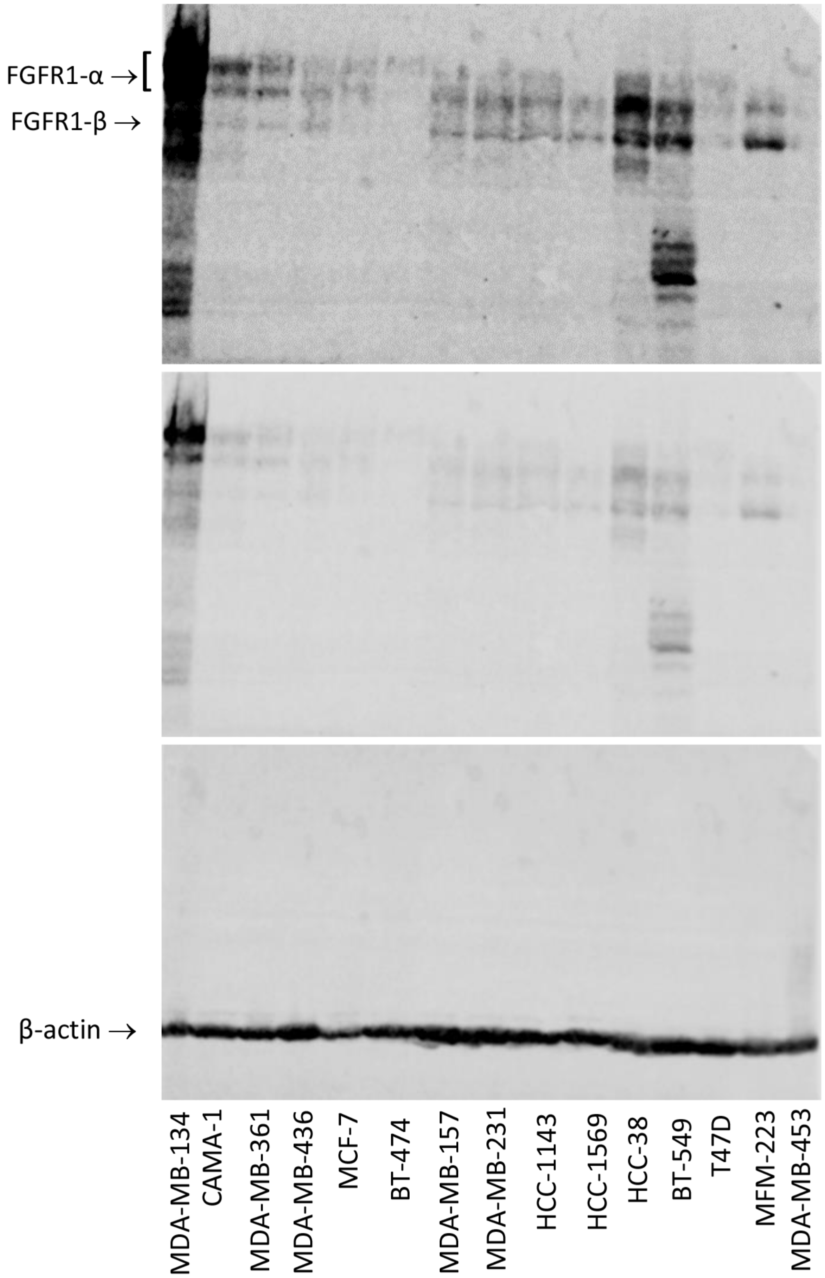

Supplementary Figure 3: Uncut immunoblotting images of Figure 1E.

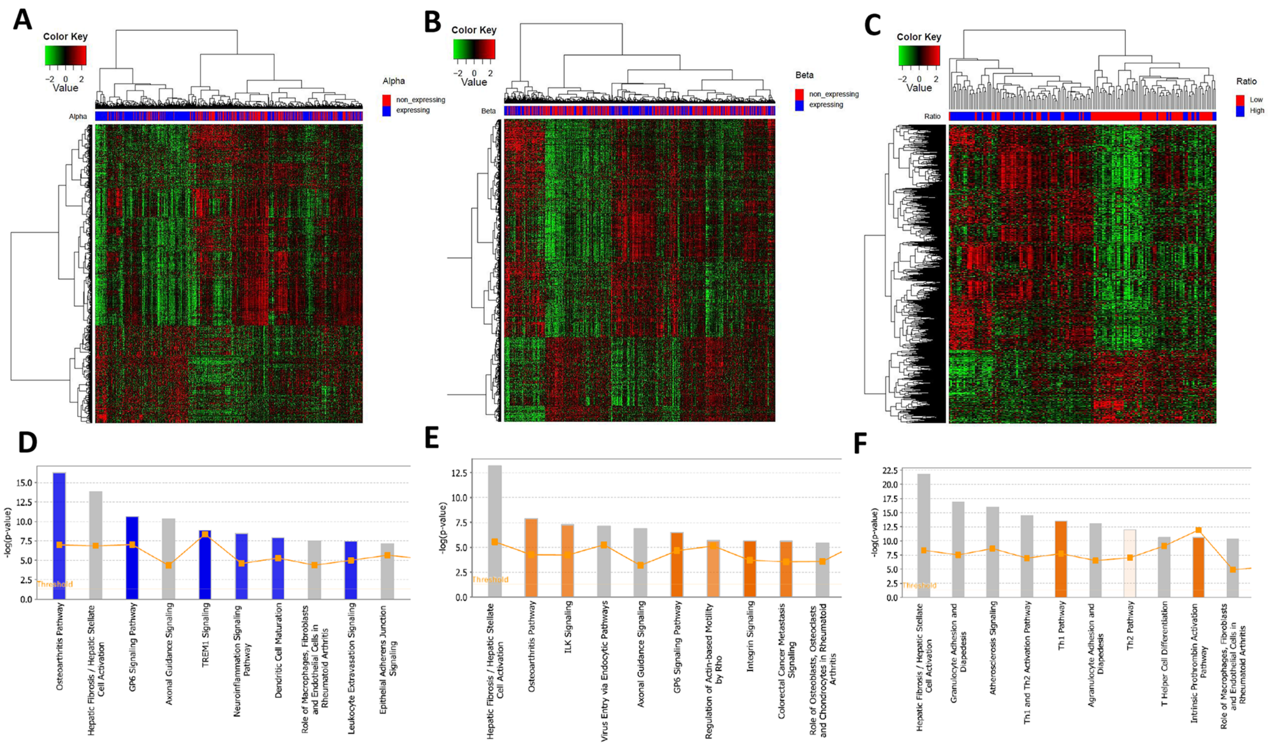

G

FDR	Significant genes α	β	β/α ratio
0.20	8286	9331	11163
0.10	6373	7340	9728
0.05	5021	5948	8524
0.01	2983	3467	6427
0.001	1512	1577	1777

Supplementary Figure 4: Analysis of DEGs and associated canonical signaling pathways. **(A-C)** Identification of differentially expressed genes (DEGs). FGFR1α-expressing and non-expressing TCGA samples (A), FGFR1β-expressing and non-expressing samples (B), and top 10% and bottom 10% samples with high and low FGFR1β/FGFR1α ratio (C), with FDR 0.001 and fold change larger than 2, were analyzed. DEGs were hierarchically clustered in heatmaps. **(D-F)** Association of canonical signaling pathways. DEGs were analyzed using Ingenuity Pathway Analysis (IPA) software (Qiagen Bioinformatics). Top 10 IPA canonical pathways were identified for each DEGs analysis between FGFR1α-expressing and non-expressing samples (D), FGFR1β-expressing and non-expressing samples (E), and top 10% and bottom 10% samples with high and low FGFR1β/FGFR1α ratio (F). The Y axis represents significance of gene enrichment for any given pathway, scored as –log (p-value) from Fisher's exact test. The yellow line refers to the number of molecules from the dataset that map to the pathway listed divided by the total number of molecules that define the canonical pathway from within the IPA knowledgebase. **(G)** Numbers of identified DEGs at different FDRs.

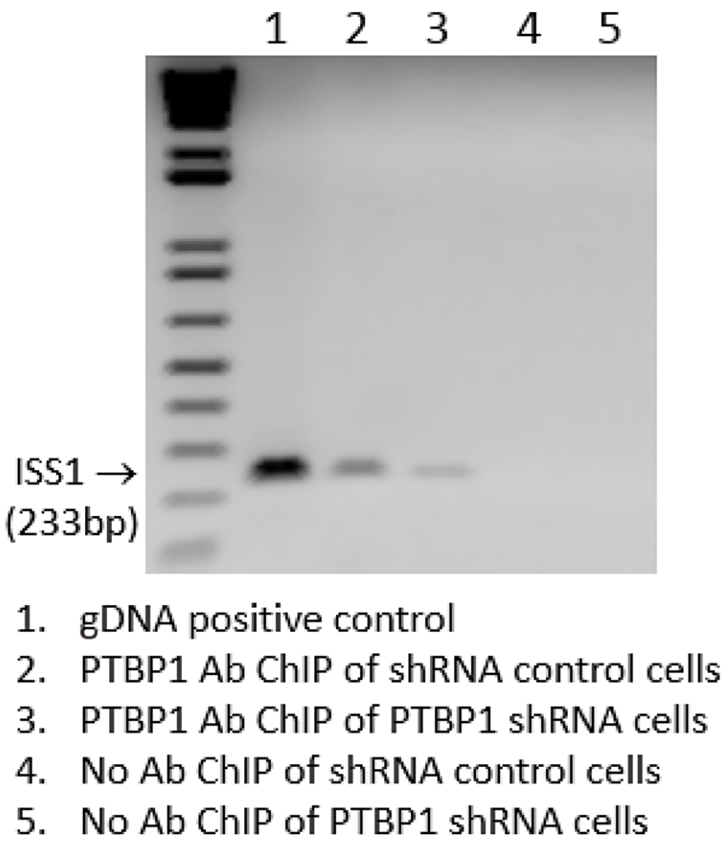

Supplementary Figure 5: ChIP assay. DNA fragments of MEM-223 cells prepared from chromatin immunoprecipitation (ChIP) with anti-PTBP1 antibody were used as templates for PCR to amplify an intronic region (233bp) in the FGFR1 gene that contains the specific PTBP1-binding intronic slicing sequence (ISS). Lane 1: positive input with gDNA; lane 2, 3: ChIP of control or PTBP1-knockdown MFM-223 cells with anti-PTBP1 antibody; lane 4, 5: ChIP without anti-PTBP1 antibody.

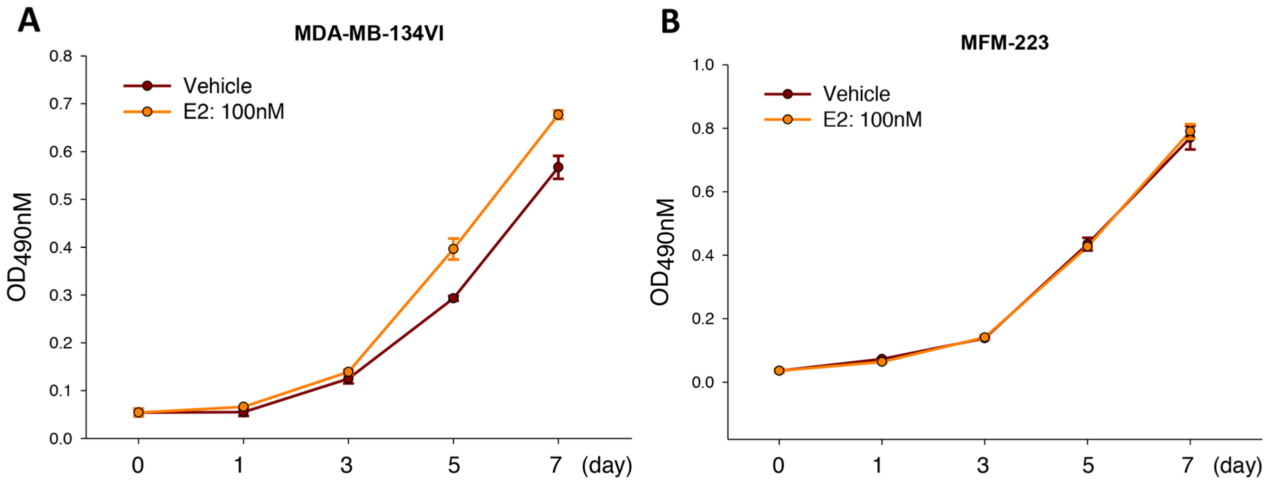

Supplementary Figure 6: Effects of estrogen on cell growth rate. MDA-MB-134VI **(A)** and MFM-223 **(B)** cells seeded in 96-well plates were incubated with 17β-estradiol at 100nM (E2) or vehicle for up to 7 days. Cell culture were stopped at the indicated time points followed by SRB staining. The plates were read at OD490nm.

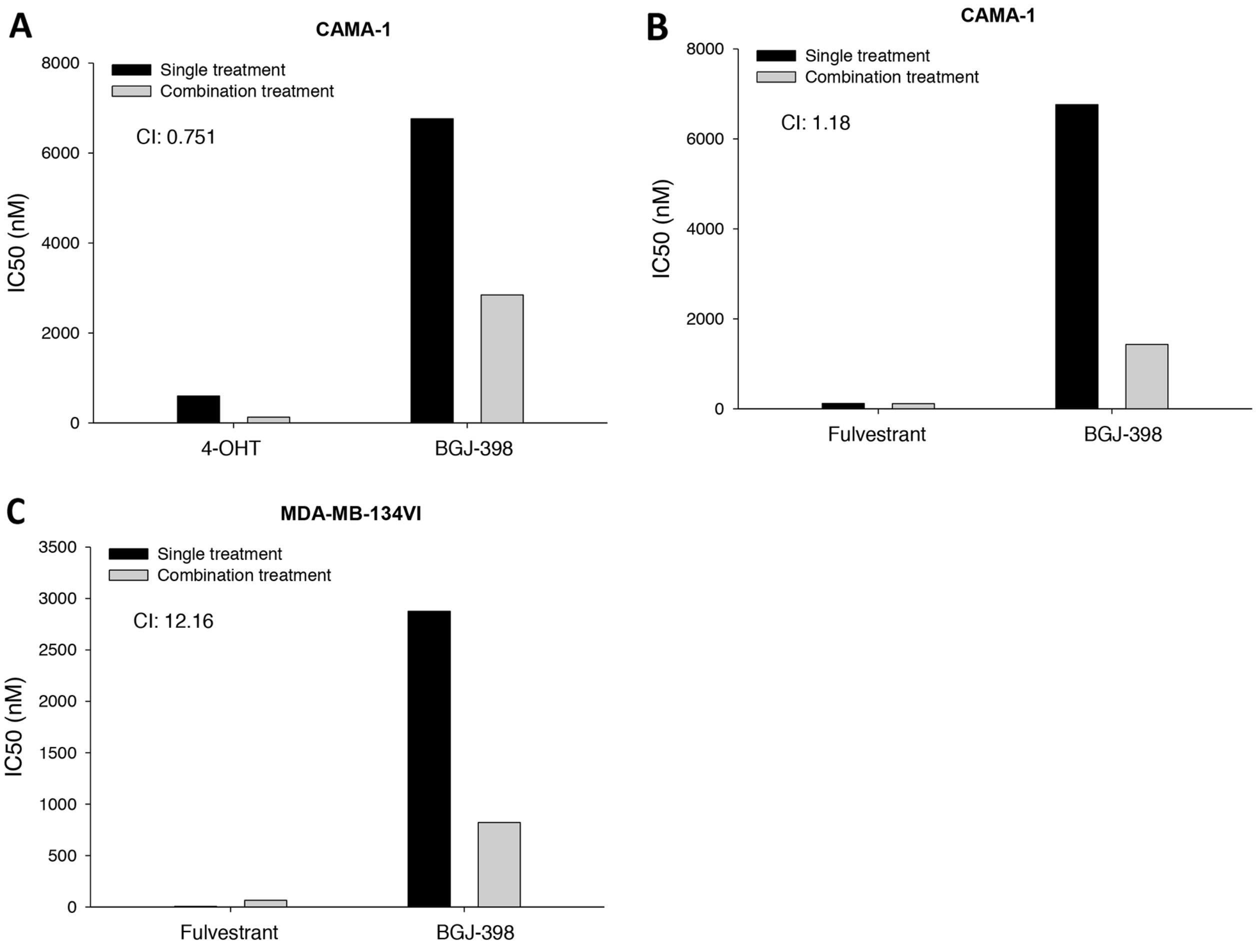

Supplementary Figure 7: Drug combination assay. CAMA-1 **(A, B)** and MDA-MB-134VI **(C)** cells seeded in 96-well plates were incubated with single or combination of 4-hydroxytamoxifen, fulvestrant, and BGJ-398 at doses from 0.05-20000nM and 1250-20000nM respectively for 5 days. Cell survival rate was measured by SRB assay. IC50 and combination index (CI) were calculated using CompuSyn software. (CI<1: synergy; CI>1: antagonism)

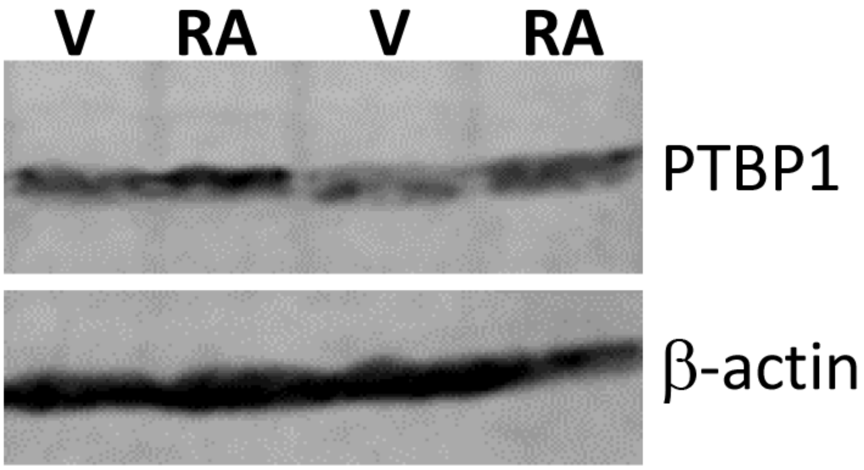

Supplementary Figure 8: Effect of retinoid acid (RA) on PTBP1 expression. MEM-223 cells were seeded into 6-well plates and treated with RA at 0.5μM or vehicle for 3 days. Cell lysates were analyzed by immunoblotting for PTBP1 expression levels with normalization by β-actin.

Supplementary Excel File: DEG FDR0.001.

See Supplementary File 1

Supplementary PDF File: IPA Pathways.

See Supplementary File 2
