# Supplementary figures and images for: FGFR1β is a driver isoform of FGFR1 alternative splicing in breast cancer cells
Source: Oncotarget. 2019 Jan 1;10(1):30–44. doi: 10.18632/oncotarget.26530 (PMC6343755; doi:10.18632/oncotarget.26530)

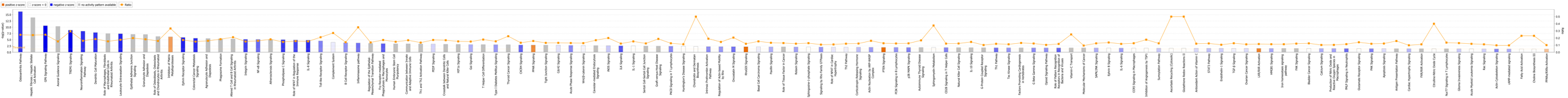

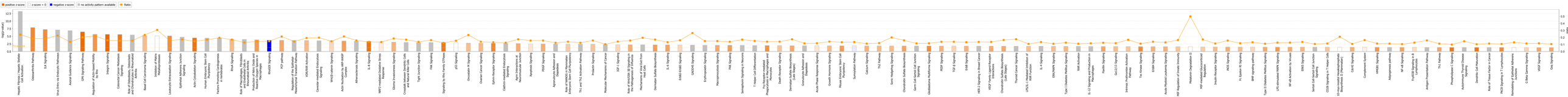

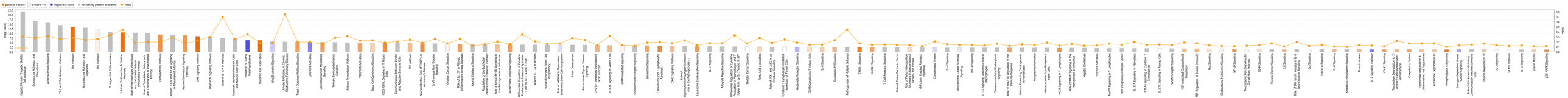

Supplement: Supplementary file 3 [file oncotarget-10-30-s003.pdf]
